# Thalidomide—Then and Now: Case Report of a Woman With Thalidomide Embryopathy and Review of Current Thalidomide Uses

**DOI:** 10.7759/cureus.17070

**Published:** 2021-08-10

**Authors:** Sara Malik, Philip R Cohen

**Affiliations:** 1 Feinberg School of Medicine, Northwestern University, Chicago, USA; 2 Dermatology, University of California, Davis Medical Center, Sacramento, USA

**Keywords:** contraception, covid-19, embryopathy, gestation, man, pregnancy, sars-cov-2, teratogen, thalidomide, woman

## Abstract

Thalidomide was initially developed as a sedative; subsequently, its use was expanded to treat morning sickness in pregnant women. However, it was later discovered to be a teratogenic drug that was associated with embryopathy in women. A woman is described who was exposed to thalidomide in utero. She had several stigmata of thalidomide embryopathy. Although treatment of nausea and anxiety in pregnant women with thalidomide was discontinued in 1961, the drug has been found to be a useful agent for the management of several systemic conditions and dermatological disorders. Whether the treatment with thalidomide shall be incorporated in the therapeutic regime for patients with severe coronavirus disease 2019 (COVID-19) infection remains to be determined.

## Introduction

Thalidomide is an anti-tumor necrosis factor-alpha drug. It was discovered in 1956 by researchers at the Chemie Grünenthal pharmaceutical company. It was released as a nonaddictive, nonbarbiturate sedative [1].

Initially, thalidomide was used by pregnant women with either nausea or anxiety, or both. In the childbearing market, its use expanded to treat insomnia in pregnant women. However, shortly thereafter, its use was associated with severe birth defects. The drug was discontinued for the treatment of pregnant women in 1961 in the United States medical market [2].

A 63-year-old woman with thalidomide embryopathy is described; her mother had been pregnant in 1957 and received the drug during her gestation. The features of thalidomide embryopathy are summarized. In addition, the current potential uses of thalidomide in medical therapy are reviewed.

## Case presentation

A 63-year-old woman presented for a total body skin examination. She had no prior history of skin cancer. However, her past medical history was significant for her mother receiving thalidomide during her pregnancy.

**Figure 1:**
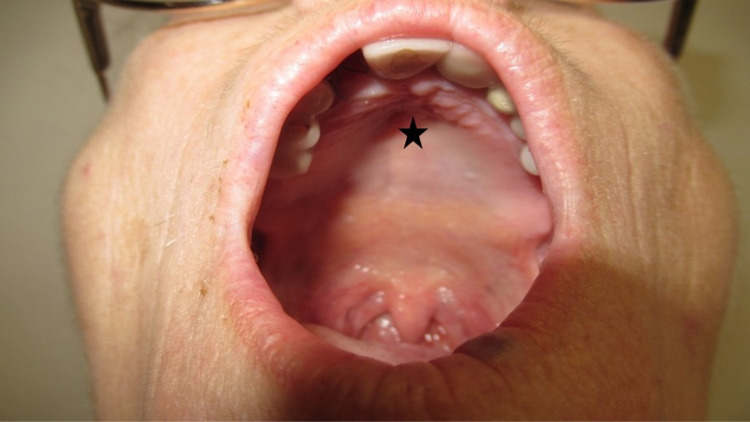
High-arched palate associated with exposure to thalidomide during gestation. Oral examination of a 63-year-old woman demonstrates thalidomide exposure-associated high-arched palate (black star).

**Figure 2:**
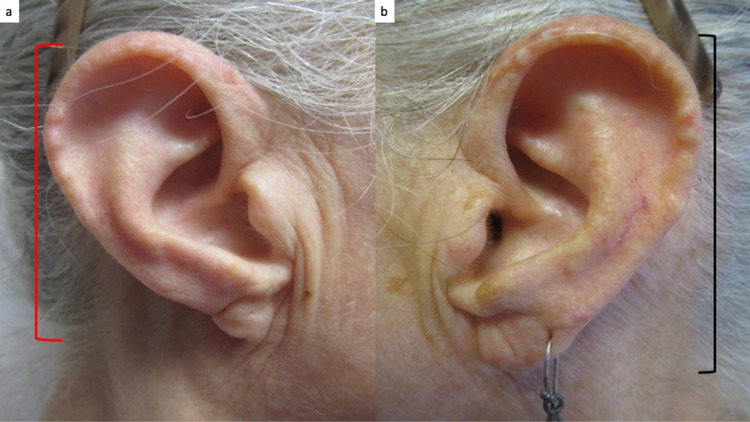
Ear abnormality in a woman with thalidomide embryopathy. The length of the external ears is different. The length of the right external ear (red bracket) measures 2.5 centimeters (a) and the length of the left ear (black bracket) measures 3 centimeters (b).

**Figure 3:**
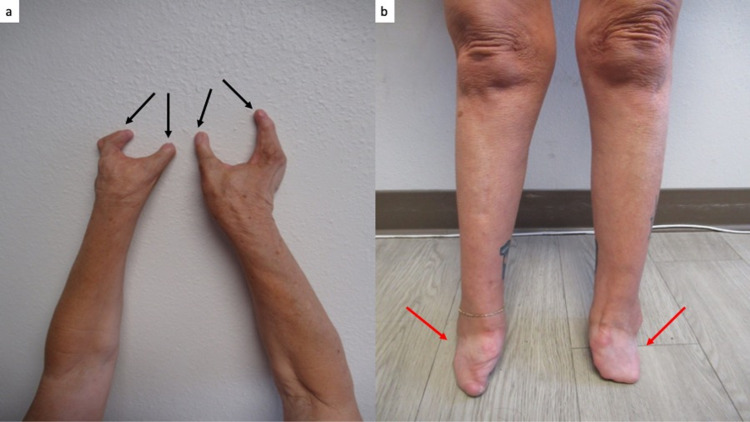
Limb deformities of the upper and lower extremities associated with thalidomide exposure during gestation. A view of the extensor surface of the arms, from the elbows to the fingers, shows that each hand only has two digits (a); the black arrows point to each digit. The anterior view of the lower extremities of the legs, from the knees to the end of the feet (red arrows); there are no toes (b). The distance from the knees to ankles is shorter than normal.

**Figure 4:**
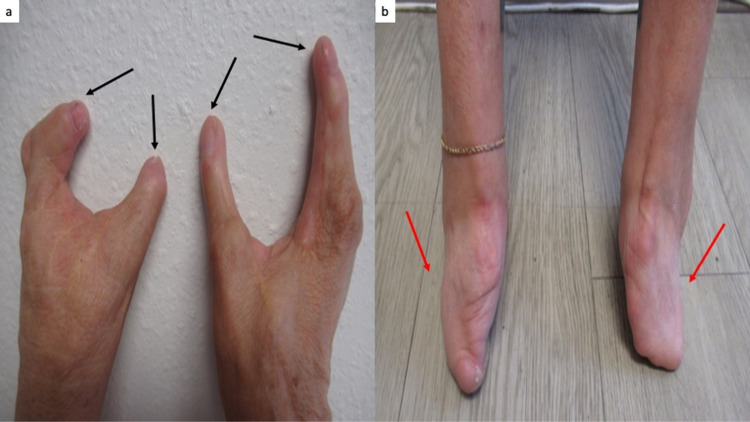
Embryopathy caused by thalidomide affecting the hands and feet. Closer views of the hands (a) and feet (b) show two digits on each hand (black arrows) and feet without toes (red arrows); she has had multiple surgeries.

She had several stigmata of thalidomide embryopathy. These included strabismus, high-arched palate, and a right external ear that was smaller than the left external ear (Figures 1, 2). She also had absent bones in her wrist, absent digits in her hands and no toes on her feet, shortened tibia and fibula, and hypermobility of her hip (Figures 3, 4).

**Figure 5:**
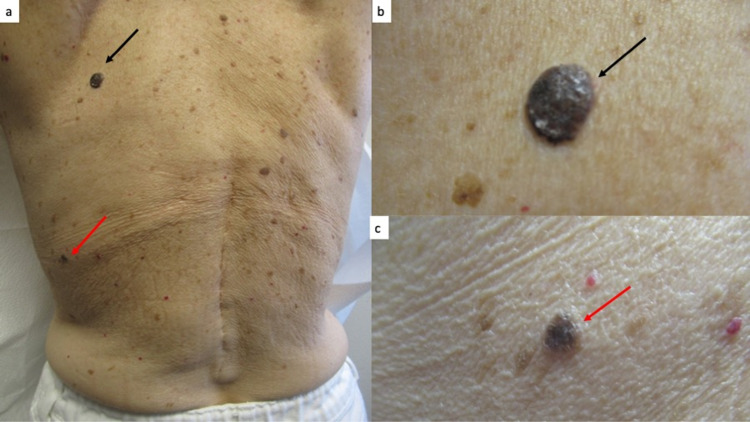
Seborrheic keratoses on the back. Distant (a) and closer (b and c) views show several black plaques on the back of a 63-year-old woman. She had noticed changes in size and color of the lesions on the upper left back (black arrow, a and b) and mid-lateral left back (red arrow, a and c). The lesions were biopsied; microscopic examination established the diagnosis of a benign irritated seborrheic keratosis for both plaques.

Cutaneous examination revealed multiple black and brown plaques on her back (Figure 5). Two of the black plaques on her back had changed in size and color; these were biopsied. Microscopic examination showed thickening of the epidermis (acanthosis) with horn cyst and heavy deposits of melanin in the basal layer of the epithelium; there was lymphocytic inflammation in the papillary dermis. Correlation of the clinical morphology and pathology findings established the diagnosis of benign irritated seborrheic keratosis.

## Discussion

Thalidomide can cause thalidomide embryopathy; this occurs in pregnant women who receive the drug as early as 20 days after fertilization. The observed adverse sequelae depend on the gestational age at the time of exposure to the drug. For example, external ear defects were noted upon thalidomide intake between day 20 and day 24 after fertilization, limb defects between 24 and 30 days after conception, hip dislocation from 24 to 34 days, and lower limb defects were noted from 27 to 34 days after fertilization [1,2].

Thalidomide exposure can cause a wide range of defects. Some of the defects that are associated with thalidomide embryopathy include phocomelia in the upper and lower limbs, reduction in the length of the lower limbs, a more prominent shoulder joint, a hypoplastic or absent hip joint, defects in the eyes, ears, and face, vertebral column defects, and damage to the internal organs [2]. Our patient had multiple hallmarks of thalidomide embryopathy affecting the limbs, hip, mouth, eye, and ear. 

Thalidomide is contraindicated in women who may become pregnant; two forms of contraception must be utilized in female patients one month prior, during, and for at least one month after discontinuing the drug. Currently, women of reproductive potential who are using the drug are registered in the THALIDOMID REMS® program. Women are required to complete a confidential survey monthly before a prescription can be written, and they must test negative in their pregnancy tests 24 hours before receiving a prescription. Furthermore, during the first four weeks, a pregnancy test needs to be performed weekly and then must be repeated every four weeks [1,2].

Contraceptive use in men who plan to be sexually active while on thalidomide is also recommended. Although sperm-associated teratogenicity has not been definitively established, male patients should use contraception not only while taking the medication but also for at least one week after stopping the drug. Indeed, despite lacking evidence on the thalidomide’s effects on male fertility, the System for Thalidomide Education and Prescribing Safety program has restricted the drug’s marketing and use in men [3].

Several systemic and dermatologic conditions may potentially be treated with thalidomide (Table 1) [4-16]. The United States Food and Drug Administration has only approved thalidomide for the treatment of erythema nodosum leprosum and multiple myeloma. In addition to thalidomide being efficacious as an anti-neoplastic agent in patients with myeloma and lymphoma, thalidomide may be an efficacious treatment for inflammatory bowel disease in patients with Crohn’s disease and possible therapeutic intervention for patients with fibrosing diseases such as pulmonary fibrosis and graft-versus-host disease. Similar to thalidomide, related analogs such as lenalidomide and pomalidomide have both been used in the treatment of cancer [4]. 

**Table 1:** Conditions potentially treated with thalidomide. Abbreviation: COVID-19, coronavirus disease 2019. ^a ^Many of the conditions treated using thalidomide have been included; however, the table is not all-inclusive. ^b ^The role of thalidomide in the treatment of COVID-19 has been postulated but not established. ^c ^United Stated Food and Drug Administration approved condition for the treatment with thalidomide. ^d ^Other conditions for which thalidomide has been used for treatment include bullous pemphigoid, histiocytosis, Jessner-Kanof disease, Kaposi sarcoma, leishmaniasis cutanea recidivans, necrobiosis lipoidica, scleromyxedema, and uremic pruritus. ^e ^A possible use of thalidomide for managing cancer cachexia has been postulated. However, the investigators of a comprehensive systematic review concluded that there was inadequate evidence to recommend thalidomide for clinical practice for patients with advanced cancer who have cachexia.

Condition^a^	References
Actinic prurigo	[4-6]
Aphthous lesions (stomatitis and ulcers)	[4-7]
Behcet disease	[5-7]
Chronic graft-versus-host disease	[6,8]
COVID-19 infection^b^	[9,10]
Crohn’s disease	[11]
Erythema multiforme	[5,6]
Erythema nodosum leprosum^c^	[4,5,7,8,11-13]
Idiopathic pulmonary fibrosis	[11]
Immune complex vasculitis	[5]
Large B-cell lymphoma	[14]
Lichen planus	[5-7]
Lupus erythematosus (chronic discoid and systemic)	[4,6,7]
Multiple myeloma^c^	[8]
Ophthalmopathy	[11]
Paraneoplastic pemphigus	[15]
Photodermatosis	[5]
Prurigo nodularis	[4-7]
Pyoderma gangrenosum	[4-7]
Rheumatoid arthritis	[5,8]
Sarcoidosis (prurigo-like lesions)	[5-7]
Scleroderma	[6]
Skin fibrosis	[11]
Other conditions^d,e^	[5,6,16]

Several mechanisms of action for thalidomide have been discovered, which include cytokine elaboration and an anti-inflammatory effect, T-cell and natural killer cell function and regulation, and anti-angiogenic properties [7,11]. Thalidomide can decrease the helper T-cell to suppressor T-cell ratio, which plays a role in the treatment of erythema nodosum leprosum. Furthermore, thalidomide’s anti-angiogenic properties are associated with its ability to treat cancer [7]. Although the exact mechanism of thalidomide is unknown, thalidomide’s different properties have allowed for its various potential uses in medical therapy. 

The role of thalidomide in coronavirus disease 2019 (COVID-19) patients has not yet been established. Some investigators have proposed that thalidomide therapy may be efficacious in patients with a severe reaction to COVID-19 infection [10,17]. A study was performed in which patients with critical COVID-19 symptoms were enrolled in the thalidomide group and control group. Analysis was performed on six patients in each group. Patients in the thalidomide group were given thalidomide (100 milligrams daily for greater than or equal to seven days) in addition to the short-term administration of low-dose dexamethasone (40 milligrams intravenously at 12-hour intervals for three days; the dose was reduced and administered at 24-hour intervals for an additional five days) while patients in the control group were given short-term low-dose dexamethasone therapy. The study showed that thalidomide accelerated the negative conversion of severe acute respiratory syndrome coronavirus 2 (SARS-CoV-2), shortened the hospital stay, and decreased the requirement for mechanical ventilation in patients with severe COVID-19. The study revealed that in COVID-19-infected patients, thalidomide with low-dose glucocorticoid therapy was efficacious in reducing inflammation, inhibiting the production of inflammatory cytokines, and enhancing modulation of the immune system [17]. 

## Conclusions

Thalidomide is a teratogenic drug that is associated with severe embryopathy when taken by pregnant women. The adverse events associated with its use during gestation are widespread. Although it was removed from the market in 1961, individuals who were exposed in utero can still be observed. Thalidomide is approved for use in leprosy patients who experience a post-treatment reaction of erythema nodosum leprosum. In addition, the drug is approved for the treatment of multiple myeloma. Moreover, there are several systemic and dermatologic conditions in which thalidomide has been demonstrated to be efficacious. Whether thalidomide has a role in the management of patients with COVID-19 remains to be established.
